# Supplementary material for: Comparative Analysis of the Effect of Inorganic and Organic Chemicals with Silver Nanoparticles on Soybean under Flooding Stress
Source: Int J Mol Sci. 2020 Feb 14;21(4):1300. doi: 10.3390/ijms21041300 (PMC7072913; doi:10.3390/ijms21041300)
Supplement: Supplementary file 1 [file ijms-21-01300-s001.zip › rev Suprimental Figures.pdf]

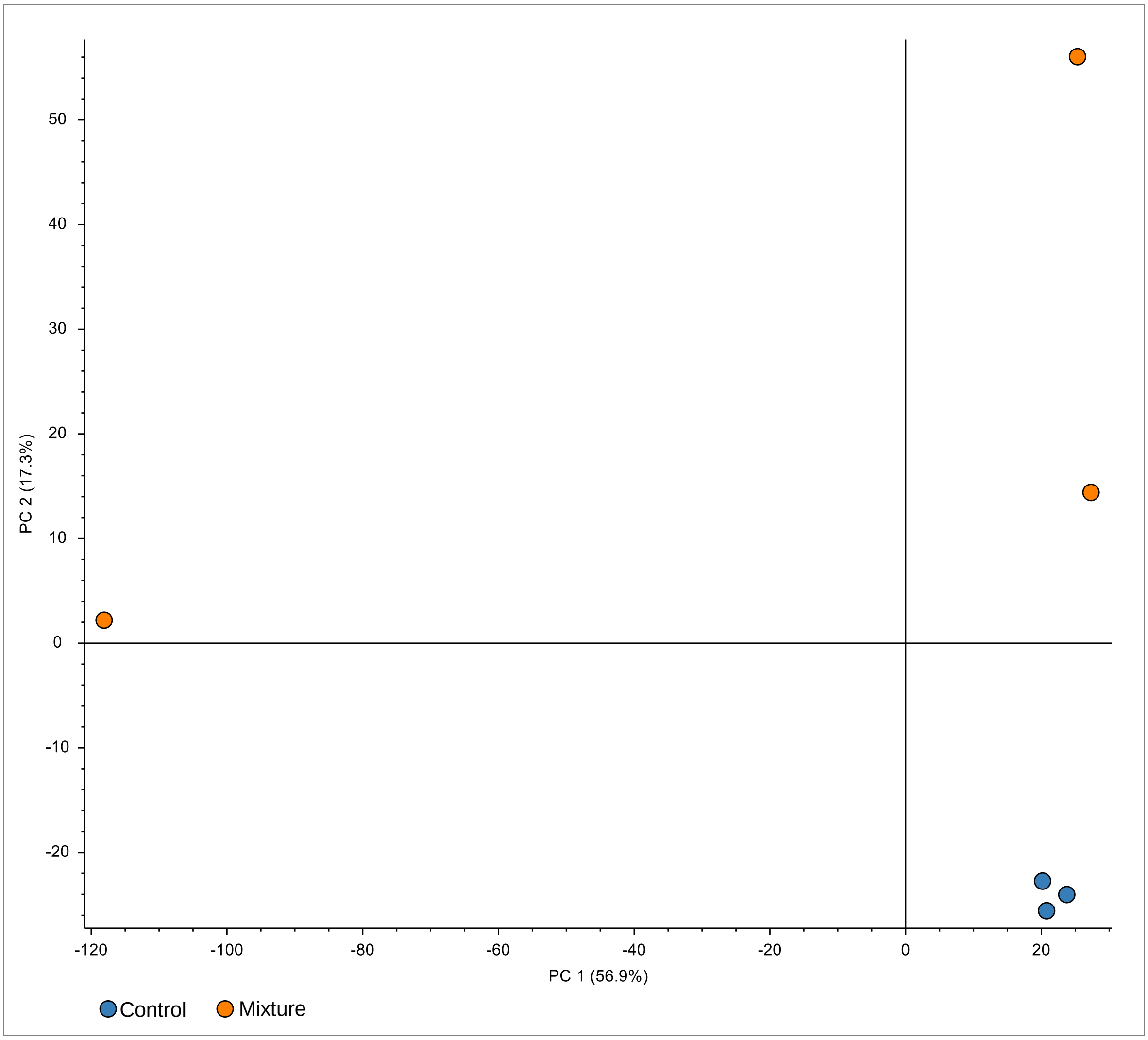

Supplemental Figure 1. Overview of data of total proteins from 6 samples based on PCA. Proteins in soybean without (Control) or with (Mixture) silver NPs and nicotinic acid/KNO_3 were extracted and analyzed using gel-free/label-free technique. Different colors of the circles indicate different groups. Three biological replicates were performed in each group.

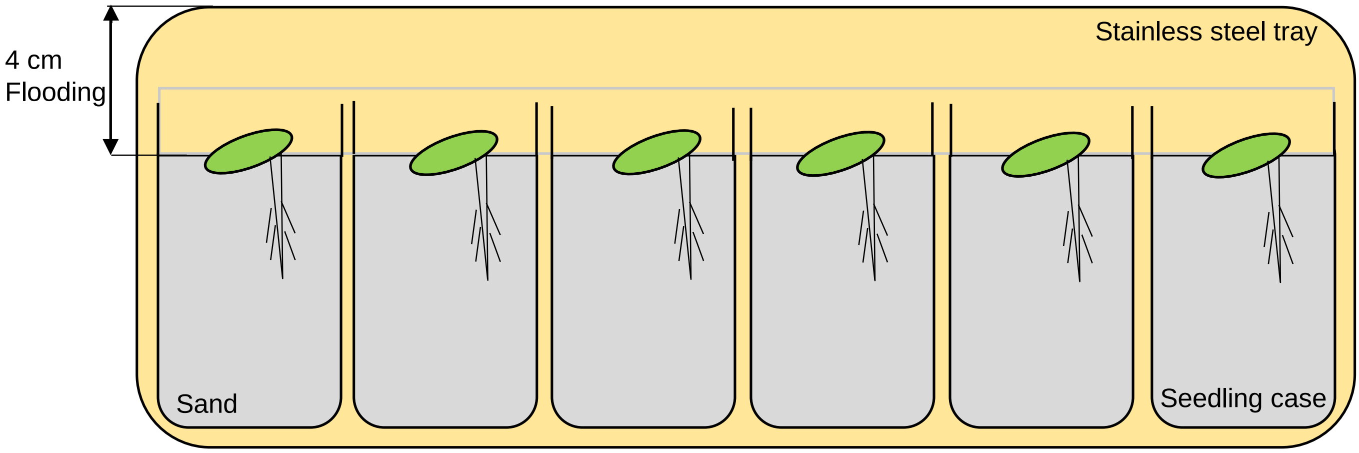

Supplemental Figure 2. Flooding treatment of soybean. Soybeans were grown in sand in plastic seedling cases. These seedling cases were kept in the stainless steel tray. Flooding treatment was performed by adding water up to 4 cm.
