# Supplementary material for: Comparative Analysis of the Effect of Inorganic and Organic Chemicals with Silver Nanoparticles on Soybean under Flooding Stress
Source: Int J Mol Sci. 2020 Feb 14;21(4):1300. doi: 10.3390/ijms21041300 (PMC7072913; doi:10.3390/ijms21041300)
Supplement: Supplementary file 1 [file ijms-21-01300-s001.zip › Supplemental Table 1.pdf]

Supplemental Table 1. List of identified proteins in flooding-stressed soybean treated with silver NPs, nicotinic acid, and KNO_3 compared to control.

No.	Accession	Description	Coverage [%]	MP[a)]	# AAs[b)]	MW [kDa][c)]	calc. pI[d)]	Ratio	*p*-Value	MapMan bin[e)]
1.	E2FKH7	Sieve element occlusion f	32	19	698	80	6.83	100	0.0000	35.2
2.	A0A0R0G789	Uncharacterized protein	43	19	499	56.1	9.01	100	0.0000	26.3
3.	I1LTQ5	AP-2 complex subunit alpha	25	17	1020	113.7	6.25	100	0.0000	31.4
4.	O81273	Biotin carboxylase	43	16	539	58.8	7.5	100	0.0000	11.1.1
5.	K7MR36	Citrate synthase	34	13	514	56.6	9.38	100	0.0000	6.1
6.	K7LST9	Uncharacterized protein	26	12	539	61.6	6.65	100	0.0000	35.1.19
7.	I1KNL7	Uncharacterized protein	48	11	409	42.3	7.93	100	0.0000	13.2.4.5
8.	A0A0R0J5W4	Cysteine synthase	46	11	389	41.3	7.93	100	0.0000	13.1.5.3.1
9.	A0A0R0KHI0	Lipoxygenase domain-containing protein	45	10	236	27.2	6.65	100	0.0000	17.7.1.2
10.	I1LXN6	Uncharacterized protein	28	10	404	46.2	6.55	100	0.0000	29.1
11.	I1LZ51	Uncharacterized protein	24	10	488	54.1	7.37	100	0.0000	13.1.3.5.5
12.	I1KP69	Uncharacterized protein	54	10	253	27.8	8.18	100	0.0000	9.1.1.5
13.	C6SVD7	Phi class glutathione S-transferase	73	10	215	24.8	6.07	100	0.0000	26.9
14.	Q5K6N6	Mitogen-activated protein kinase	38	10	391	44.8	5.86	100	0.0000	30.6
15.	I1LHP2	Uncharacterized protein	31	9	382	43.2	5.68	100	0.0000	29.1.1
16.	K7MJR6	Uncharacterized protein	19	9	803	91	6.51	100	0.0000	35.2
17.	I1J8S9	Uncharacterized protein	61	8	213	24.1	5.34	100	0.0000	23.4.99
18.	K7KQ62	FMN hydroxy acid dehydrogenase domain-containing protein	29	8	298	32.3	9.28	100	0.0000	1.2.2
19.	K7KEG4	Uncharacterized protein	24	7	483	54.8	8.24	100	0.0000	17.3.1.2.3
20.	I1KZI6	ENTH domain-containing protein	10	6	922	98.5	5.87	100	0.0000	35.1.21
21.	I1M393	Thioredoxin domain-containing protein	28	5	182	19.6	8.94	100	0.0000	21.1
22.	D3G9M7	Calcium dependent protein kinase	11	5	579	64.9	5.43	100	0.0000	30.3
23.	I1M4Q3	Uncharacterized protein	6	5	982	110.1	7.55	100	0.0000	30.11
24.	C6TCW7	GrpE protein homolog	27	5	290	32.4	5.59	100	0.0000	29.6
25.	I1JG92	Uncharacterized protein	8	4	759	86.3	6.23	100	0.0000	35.2
26.	K7LM54	Rab-GAP TBC domain-containing protein	7	4	830	93.2	4.83	100	0.0000	30.5
27.	I1KXP5	A deaminase domain-containing protein	7	4	866	99	6.3	100	0.0000	23.2
28.	I1JLR9	Uncharacterized protein	8	4	886	98.7	5.02	100	0.0000	33.99
29.	K7K2V9	MI domain-containing protein	3	4	1822	196.5	7.36	100	0.0000	29.2.3

30.	K7MU41	Uncharacterized protein	9	4	596	66.9	8.53	100	0.0000	20.2.3
31.	A0A0R0IQJ4	Uncharacterized protein	15	4	285	32.6	7.87	100	0.0000	26.9
32.	I1J7U8	Epimerase domain-containing protein	10	3	438	48.6	9.85	100	0.0000	10.1.6
33.	A0A0R0J7H3	RmlD sub bind domain-containing protein	12	3	350	38.9	8.69	100	0.0000	35.1
34.	K7KW60	Uncharacterized protein	14	3	295	31.8	7.69	100	0.0000	13.1.5.1.3
35.	I1MPF0	Glycosyltransferase	7	3	473	52.2	6.14	100	0.0000	26.2
36.	K7MP06	Uncharacterized protein	2	3	1197	137	6.42	100	0.0000	27.1
37.	I1MDR1	Uncharacterized protein	10	3	516	58.2	6.46	100	0.0000	35.1
38.	I1J4W0	Uncharacterized protein	19	3	221	24.3	5.99	100	0.0000	35.2
39.	C6TII4	Serine/threonine-protein phosphatase	13	3	316	35.9	5.36	100	0.0000	29.4
40.	I1KAG4	Putative methyl transferase	6	3	835	93.6	5.26	100	0.0000	20.2.3
41.	I1LBY1	Protein kinase domain-containing protein	7	3	700	78	6.57	100	0.0000	29.4
42.	A0A368UKK5	Peroxidase	12	3	518	57.2	7.69	100	0.0000	30.5
43.	I1MRW2	Importin N-terminal domain-containing protein	3	2	1110	123.7	4.83	100	0.0000	29.3.1
44.	I1J975	Uncharacterized protein	12	2	174	19.6	8.57	100	0.0000	30.8
45.	I1N5E0	Uncharacterized protein	3	2	1118	123	9	100	0.0000	35.2
46.	A0A0R0GCU0	PHD-type domain-containing protein	2	2	1253	138.2	6.7	100	0.0000	33.99
47.	C6TIW1	Uncharacterized protein	8	2	301	32.5	6.15	100	0.0000	35.1
48.	I1M926	Cytosolic Fe-S cluster assembly factor NBP35	8	2	355	38.2	4.94	100	0.0000	18.7
49.	C6SZF0	AAI domain-containing protein	20	2	93	10	8.87	100	0.0000	26.21
50.	I1KS28	Uncharacterized protein	10	2	96	11	9.38	100	0.0000	35.2
51.	I1LYG4	t-SNARE coiled-coil homology domain-containing protein	7	2	269	30.3	6.58	100	0.0000	31.4
52.	I1K559	Uncharacterized protein	3	2	687	79.3	6.62	100	0.0000	27.3.99
53.	K7KYQ1	SUN domain-containing protein	4	2	605	69	6	100	0.0000	35.2
54.	I1M7R6	Uncharacterized protein	4	2	535	59.7	6.92	100	0.0000	35.1
55.	I1J8T9	Gluce-6-phosphate 1-epimerase	6	2	311	33.8	5.88	100	0.0000	3.5
56.	K7M3M7	Uncharacterized protein	9	2	333	35	7.77	100	0.0000	7.1.3
57.	I1N123	PsbP domain-containing protein	7	2	265	28.4	7.87	100	0.0000	1.1.1.2
58.	C6TL77	Uncharacterized protein	9	2	262	29.8	6.93	100	0.0000	27.2
59.	C6TME8	Uncharacterized protein	12	2	220	25.1	9.01	100	0.0000	31.4
60.	I1K0I8	PPC domain-containing protein	13	2	327	33.4	8.95	100	0.0000	27.3.67

61.	I1LP93	Phospholipid scramblase	7	2	349	40.2	9.57	100	0.0000	35.1
62.	I1KIH5	Methyl transferase	5	2	641	73.1	8.16	100	0.0000	20.2.3
63.	I1K2W9	FAD-binding PCMH-type domain-containing protein	3	2	536	60.7	9.2	100	0.0000	26.8
64.	I1K665	Trehalase	4	2	580	65.7	5.86	100	0.0000	3.2.4
65.	I1MK29	Uncharacterized protein	6	2	416	45.2	7.78	100	0.0000	27.1.1
66.	I1JD34	Uncharacterized protein	5	2	472	51.2	8.32	100	0.0000	23.2
67.	C6T1L7	Bet v 1 domain-containing protein	20	2	155	17.6	8.12	100	0.0000	20.2.99
68.	K7KUR7	RRM domain-containing protein	3	2	1176	128.8	6.29	49.322	0.0000	29.5.7
69.	I1JMM6	Uncharacterized protein	25	7	445	48.9	5.16	29.168	0.0000	31.1
70.	I1N5X2	Peroxidase	35	7	323	35.5	8.29	14.952	0.0000	26.12
71.	I1KQU5	Malate dehydrogenase	28	4	356	37.4	8.29	14.739	0.0000	6.3
72.	A0A0R0J569	Uncharacterized protein	5	2	422	46	7.78	12.666	0.0000	27.1.1
73.	C6SX42	Glutaredoxin domain-containing protein	27	3	166	17.9	9.07	9.941	0.0000	21.4
74.	I1MLU4	Uncharacterized protein	17	4	372	38.8	7.66	9.719	0.0000	27.4
75.	K7N1D8	AMP-binding domain-containing protein	39	17	725	79.7	7.23	8.88	0.0000	11.1.9
76.	K7KJI6	TPR REGION domain-containing protein	5	2	738	82.2	4.81	8.484	0.0000	29.3.5
77.	I1L1F3	Uncharacterized protein	50	11	242	28.3	9.83	8.363	0.0000	29.2.1.2.2.7
78.	I1L280	Uncharacterized protein	26	3	150	17.1	7.81	7.672	0.0000	9.1.1
79.	K7MK51	Uncharacterized protein	13	6	817	87.6	6.98	7.135	0.0000	29.5.1
80.	K7KYV7	Lipoxygenase domain-containing protein	34	5	170	19.2	8.94	6.338	0.0000	17.7.1.2
81.	I1LSY9	Usp domain-containing protein	27	6	256	27.6	6.19	6.096	0.0000	20.2.99
82.	I1KMX8	Uncharacterized protein	28	2	121	12.9	4.78	5.944	0.0000	35.2
83.	C6TCX5	Peptidase M24 domain-containing protein	49	13	394	43.5	6.7	5.817	0.0000	29.5
84.	I1NFG9	Uncharacterized protein	52	18	585	65.5	5.07	5.094	0.0000	29.4
85.	I1NED8	Uncharacterized protein	1	2	1296	134.5	8.78	5.091	0.0000	35.2
86.	I1K4M6	C2 domain-containing protein	17	23	2151	229.2	5.36	5.018	0.0000	35.1.19
87.	I1KY46	Uncharacterized protein	6	3	465	52.3	8.44	4.966	0.0000	16.2
88.	I1M3U3	AB hydrolase-1 domain-containing protein	15	4	311	34.7	5.29	4.816	0.0000	26.1
89.	I1MXX5	Proteasome subunit alpha type	60	11	284	31	5.19	4.496	0.0000	29.5.11.20
90.	Q948P6	Ferritin-3, chloroplastic	27	5	256	28.9	5.52	4.322	0.0000	15.2
91.	A0A0R0HPA5	Uncharacterized protein	39	8	216	24.7	6.54	4.276	0.0000	23.4.99

92.	I1M290	KOW domain-containing protein	29	4	146	16.7	10.92	4.178	0.0000	29.2.1.2.2.26
93.	I1MAV1	Glycosyltransferase	9	2	488	54.5	5.54	4.123	0.0000	26.2
94.	I1LC70	GLTP domain-containing protein	22	4	202	22.5	6.9	4.091	0.0000	11.6
95.	I1M675	Uncharacterized protein	7	3	686	76.6	5.55	4.016	0.0000	31.1
96.	I1LVY0	methyl transferase	6	3	694	78.9	6.06	3.98	0.0000	20.2.3
97.	C6T2F1	UBIQUITIN CONJUGAT 2 domain-containing protein	30	2	148	16.5	6.92	3.973	0.0000	29.5.11.3
98.	I1K6Z1	HECT domain-containing protein	5	6	1895	204.1	5.82	3.791	0.0001	29.5.11.4.1
99.	I1K5C0	TPR REGION domain-containing protein	14	6	587	63.8	8.87	3.724	0.0000	29.3.3
100.	I1NEH6	Aldo ket red domain-containing protein	66	13	328	36.6	7.74	3.412	0.0000	34.15
101.	I1JCF9	VWFA domain-containing protein	37	10	405	43	4.67	3.366	0.0001	29.5.11
102.	I1MLV5	Fe2OG dioxygenase domain-containing protein	28	6	312	35.8	5.69	3.215	0.0000	26.14
103.	I1K6Q2	Uncharacterized protein	8	2	433	48.3	5.78	3.19	0.0000	35.1
104.	I1L0D9	Peroxidase	32	6	349	38	7.3	3.025	0.0000	26.12
105.	K7MVS5	Protein kinase domain-containing protein	11	4	671	75.2	6.7	2.885	0.0000	29.4
106.	I1LGL4	Uncharacterized protein	22	5	410	45.1	7.2	2.883	0.0000	35.1
107.	I1JKM6	Uncharacterized protein	31	5	289	32.6	5.29	2.845	0.0000	#N/A
108.	C6T482	Uncharacterized protein	54	2	89	10.6	9.35	2.841	0.0000	35.2
109.	I1MJI9	Citrate synthase	58	18	472	52.5	8.47	2.806	0.0000	8.1.2
110.	I1K0S4	Uncharacterized protein	9	2	281	30.5	7.09	2.69	0.0000	35.2
111.	I1JKU5	MSP domain-containing protein	20	4	241	26.7	7.75	2.664	0.0008	29.3.4.1
112.	I1JDM8	W2 domain-containing protein	12	4	441	48.7	6.64	2.654	0.0019	29.2.3
113.	I1MKY3	Uncharacterized protein	27	2	112	12.6	6.74	2.653	0.0000	#N/A
114.	I1MXJ1	Uncharacterized protein	25	10	777	82.2	7.09	2.636	0.0000	29.5.1
115.	I1MJ28	Uncharacterized protein	62	29	591	62.8	5.87	2.564	0.0000	29.6
116.	I1LES5	PB1 domain-containing protein	5	3	707	76.5	6.18	2.531	0.0139	35.1
117.	I1M3W1	Ribosomal S7 domain-containing protein	51	10	204	22.6	9.48	2.526	0.0000	29.2.1.2.1.5
118.	I1KBY3	UVR domain-containing protein	53	36	922	102.4	6.46	2.486	0.0020	29.5.5
119.	I1JWV7	Uncharacterized protein	43	8	262	30.2	4.31	2.483	0.0002	28.1
120.	C6TK28	Serine/threonine-protein phphatase	54	10	303	34.8	5.01	2.403	0.0000	29.4
121.	I1L738	Peroxidase	10	2	322	34.2	5.27	2.368	0.0003	26.12
122.	I1M8S3	Uncharacterized protein	27	11	774	82.1	7.39	2.349	0.0000	29.5.1

123.	I1MY43	Vacuolar protein sorting-associated protein 35	4	2	798	90.3	5.43	2.326	0.0231	29.3.4.3
124.	K7KLB5	Uncharacterized protein	18	7	557	62.4	4.88	2.288	0.0361	34.99
125.	I1L2J3	Uncharacterized protein	7	3	600	66.7	7.18	2.287	0.0000	28.1
126.	I1JK28	FSH1 domain-containing protein	19	4	268	30.1	6.24	2.273	0.0205	35.2
127.	I1N2G8	Uncharacterized protein	56	2	55	5.8	4.32	2.164	0.0015	15.2
128.	C6TDZ1	Uncharacterized protein	53	6	286	30.7	6.67	2.143	0.0000	34.19.1
129.	I1JUI8	PsbP domain-containing protein	6	2	298	34.3	7.24	2.128	0.0034	1.1.1.2
130.	C6SZJ5	FAS1 domain-containing protein	14	2	245	26.3	9.57	2.061	0.0000	10.5.1
131.	I1JBK4	Uncharacterized protein	32	3	160	17.5	5.19	2.023	0.0002	35.2
132.	C6SVL0	Uncharacterized protein	21	3	200	21.8	5.33	2.012	0.0000	20.1.7.6.1
133.	I1NGL3	Uncharacterized protein	32	11	466	52.2	7.3	2.009	0.0039	14.1
134.	A0A0R0JSZ6	Ferritin	36	2	89	10.2	5.69	2.008	0.0005	15.2
135.	I1L776	Uncharacterized protein	29	6	357	39.9	5.29	1.987	0.0074	16.2.1.9
136.	C6SW49	Uncharacterized protein	30	2	125	13.5	7.97	1.987	0.0260	35.2
137.	I1JKM1	Uncharacterized protein	45	9	289	32.3	5.17	1.942	0.0000	#N/A
138.	I1JAN2	Uncharacterized protein	10	6	859	97.1	4.88	1.94	0.0115	35.1
139.	C6T034	EF1 GNE domain-containing protein	76	14	223	24.3	4.68	1.914	0.0000	29.2.4
140.	I1K4S0	Uncharacterized protein	57	13	241	27.5	6.44	1.887	0.0015	9.9
141.	A0A0R0FQV9	Xyloglucan endotransglucosylase/hydrolase	40	13	290	33.7	6.93	1.869	0.0000	10.7
142.	Q2LAL4	Cytochrome P450 monooxygenase CYP83E8	43	14	499	57.4	7.96	1.841	0.0000	16.5.1.1.4.1
143.	A0A0R0FHG0	AAI domain-containing protein	34	3	95	9.9	8.41	1.838	0.0000	26.21
144.	C6TAH8	Uncharacterized protein	49	11	276	29.1	9.11	1.832	0.0058	34.2
145.	I1K672	Importin N-terminal domain-containing protein	41	21	870	96.3	4.78	1.814	0.0001	29.3.1
146.	I1L377	Uncharacterized protein	19	3	206	22.5	5.82	1.805	0.0002	27.3.99
147.	A0A0R0K0E5	FAD-binding PCMH-type domain-containing protein	8	4	530	59.2	7.85	1.803	0.0049	26.8
148.	I1K1P3	Uncharacterized protein	8	2	366	39.6	8.98	1.795	0.0043	13.2.3.4
149.	I1MGH2	Fn3 like domain-containing protein	18	9	775	84	8.15	1.78	0.0000	10.6.2
150.	C6T2H1	Flavodoxin-like domain-containing protein	60	9	203	21.7	6.95	1.776	0.0025	11.8
151.	A0A0R0KVX2	Fe2OG dioxygenase domain-containing protein	18	4	349	39.7	6.84	1.697	0.0001	35.1
152.	A0A0R0GAQ0	Peptidase A1 domain-containing protein	7	3	472	50.1	8.75	1.692	0.0000	27.3.99
153.	K7MPG5	Uncharacterized protein	28	6	389	42.4	8.29	1.689	0.0006	13.1.1.3.11

154.	A0A0R4J4M3	FAS1 domain-containing protein	13	2	262	27.8	8.88	1.689	0.0010	10.5.1
155.	I1KW34	Uncharacterized protein	29	8	415	45	5.02	1.677	0.0113	29.5
156.	I1JEY3	Fe2OG dioxygenase domain-containing protein	22	3	352	40.8	6.37	1.67	0.0030	21.2
157.	I1LTS0	Arogenate dehydratase	10	3	425	46.2	7.84	1.67	0.0180	13.1.6.3.1
158.	I1JPW6	Uncharacterized protein	13	3	314	36.2	9.44	1.653	0.0008	27.1.1
159.	C6SYN6	N-acetyltransferase domain-containing protein	12	2	165	18.6	8.6	1.645	0.0221	26.24
160.	C6TIC3	Uncharacterized protein	50	9	286	30.7	8.65	1.644	0.0001	34.19.1
161.	I1MRM5	Bet v 1 domain-containing protein	55	6	157	17.1	5.07	1.638	0.0001	20.2.99
162.	I1MNV4	t-SNARE coiled-coil homology domain-containing protein	10	2	308	34.8	7.94	1.617	0.0294	31.4
163.	A0A0R0F259	Uncharacterized protein	46	17	608	65.2	6.21	1.61	0.0013	5.2
164.	I1LZR2	AAI domain-containing protein	19	3	227	23.5	8.79	1.609	0.0005	26.21
165.	I1N4U1	6-phosphogluconate dehydrogenase, decarboxylating	69	27	486	53.6	5.9	1.606	0.0004	7.1.3
166.	A0A0R0INP8	Peroxidase	29	8	321	34.5	8.9	1.596	0.0000	26.12
167.	K7LE13	Abhydrolase 3 domain-containing protein	12	3	324	36.6	5.11	1.593	0.0056	35.1
168.	I1JSR6	Uncharacterized protein	13	3	349	39.2	6.04	1.591	0.0046	29.5.3
169.	C6SXK8	Phytocyanin domain-containing protein	32	2	121	12.9	9.5	1.59	0.0000	26.19
170.	I1N5H2	Uncharacterized protein	78	12	181	20.6	6.95	1.588	0.0362	29.4
171.	C6T7F3	PfkB domain-containing protein	67	13	341	37.5	5.47	1.578	0.0000	23.3.2.1
172.	C6TLJ6	Uncharacterized protein	67	10	287	30.8	8.13	1.575	0.0318	34.19.1
173.	I1JRU5	Lipase GDSL domain-containing protein	25	6	365	40.4	5.74	1.563	0.0004	26.28
174.	K7MAQ4	DUF3700 domain-containing protein	23	5	293	32.1	6.79	1.562	0.0015	15
175.	A0A0R0F156	Uncharacterized protein	25	4	265	29.4	5.4	1.56	0.0053	26.28
176.	I1MG21	Peroxidase	19	4	349	37.6	5.82	1.559	0.0007	26.12
177.	C6SZA9	Bet v 1 domain-containing protein	63	9	153	17.1	5.25	1.554	0.0003	20.2.99
178.	A0A0R0I2M6	Peroxidase	38	8	355	38.5	6.04	1.531	0.0002	26.12
179.	C6SZ93	AAI domain-containing protein	22	2	95	9.9	8.57	1.531	0.0008	26.21
180.	K7LKJ0	HMA domain-containing protein	48	3	81	8.6	6.6	1.527	0.0017	15.2
181.	A0A0R0G0H4	Uncharacterized protein	38	5	285	30.4	8.15	1.521	0.0028	34.19.1
182.	C6SZQ8	Phytocyanin domain-containing protein	26	2	168	17.3	6.76	1.52	0.0198	26.19
183.	A0A0R0KTP5	Uncharacterized protein	38	5	285	30.4	8.15	1.517	0.0292	34.19.1
184.	I1J4B5	Uncharacterized protein	16	4	462	51	6.68	1.517	0.0378	23.4.99

185.	C6SXM4	Uncharacterized protein	78	13	181	20.7	6.95	1.516	0.0007	29.3.4.99
186.	I1KBS7	Uncharacterized protein	32	10	557	62.5	4.86	1.491	0.0222	34.99
187.	K7MNX9	Beta-galactosidase	29	13	732	81.7	8.4	1.488	0.0014	26.3.2
188.	K7N2G0	Uncharacterized protein	25	10	787	85.8	4.37	1.487	0.0106	35.1
189.	A0A0R4J3D2	Sm domain-containing protein	33	2	99	10.7	4.84	1.476	0.0036	27.1
190.	I1JS35	FAD-binding PCMH-type domain-containing protein	34	14	595	65.5	7.2	1.475	0.0017	35.1
191.	I1LPL0	FAD-binding PCMH-type domain-containing protein	52	21	567	66	8.22	1.471	0.0053	17.3.1.2.8
192.	I1N3Z3	Nudix hydrolase domain-containing protein	42	11	301	34	6.05	1.468	0.0383	16.1.2.7
193.	I1KEH1	Phytocyanin domain-containing protein	20	3	217	23.2	7.87	1.463	0.0323	26.19
194.	C6SXW7	Uncharacterized protein	29	6	207	23.1	4.93	1.46	0.0011	30.4
195.	C6T265	Ferredoxin	50	3	154	16.5	5.88	1.46	0.0259	7.3
196.	K7LJD7	Uncharacterized protein	40	20	953	104.2	4.3	1.457	0.0029	35.1
197.	I1N5R9	Formate dehydrogenase, mitochondrial	72	19	375	41.3	7.34	1.452	0.0001	25.1
198.	A0A0R0KYU7	Xyloglucan endotransglucylase/hydrolase	45	15	309	36	8.25	1.452	0.0001	10.7
199.	I1L090	Endoglucanase	11	5	618	68.3	8.68	1.44	0.0001	10.2
200.	A0A0R4J307	Uncharacterized protein	17	3	250	25.4	6.51	1.439	0.0001	34.19.2
201.	C6SVX9	HMA domain-containing protein	82	5	130	13.6	4.7	1.426	0.0098	15.2
202.	A0A0R0KKE2	Uncharacterized protein	28	3	106	11.8	8.87	1.426	0.0435	17.6.3
203.	I1KUQ6	Lipoxygenase	29	18	868	97.1	7.4	1.417	0.0072	17.7.1.2
204.	I1KHB7	Germin-like protein	46	3	225	23.8	8.46	1.415	0.0018	20.2.99
205.	I1KYX1	Uncharacterized protein	52	10	212	23.4	5.05	1.414	0.0003	20.1.7.6.1
206.	I1KC33	Purple acid phosphatase	41	20	623	70.4	6.23	1.413	0.0003	26.13
207.	I1LKM5	Pyr redox 2 domain-containing protein	43	14	360	39.3	9.42	1.409	0.0003	35.1
208.	I1LP66	Uncharacterized protein	18	12	850	96.7	4.82	1.409	0.0062	35.1
209.	I1J7T0	Pectinesterase	16	6	515	56.5	9.01	1.406	0.0325	10.8.99
210.	I1KDM8	Malate dehydrogenase	71	15	345	36	8.1	1.404	0.0048	8.1.9
211.	A0A0R4J629	Uncharacterized protein	17	3	250	25.5	6.51	1.402	0.0025	34.19.2
212.	I1J7B3	Uncharacterized protein	26	7	398	43.4	6.24	1.4	0.0156	26.11.1
213.	I1M9K9	Uncharacterized protein	33	9	366	39.8	7.74	1.399	0.0183	35.2
214.	C6SX81	Ferredoxin	44	4	144	15.5	4.77	1.398	0.0209	1.1.5.2
215.	I1LUM3	14 3 3 domain-containing protein	68	14	260	29.3	4.82	1.394	0.0262	30.7

216.	K7LNG5	Isoflavone reductase	56	13	318	35.6	5.73	1.38	0.0271	16.8.5
217.	A0A0R4J2V8	Dirigent protein	18	3	215	23.9	6.28	1.376	0.0264	20.1.7
218.	I1JQQ6	J domain-containing protein	22	5	343	38.8	6.74	1.372	0.0317	20.2.1
219.	A0A368UH97	Uncharacterized protein	26	12	568	63.9	7.68	1.368	0.0338	#N/A
220.	I1MK76	Pectinesterase	30	11	492	54.7	7.75	1.365	0.0498	10.8.1
221.	I1KPN5	Uncharacterized protein	50	33	708	78	5.24	1.358	0.0175	20.2.1
222.	I1MNK0	Uncharacterized protein	47	13	331	35	7.06	1.356	0.0414	1.1.1.2
223.	I1L1Q3	Uncharacterized protein	18	4	406	45.1	7.2	1.355	0.0419	29.5.3
224.	A0A368UGT8	Uncharacterized protein	53	13	463	50.4	5.14	1.351	0.0029	23.2
225.	C6ZS03	Leucine rich repeat protein	53	17	368	39.7	8.46	1.348	0.0032	35.1
226.	I1LWI3	PHB domain-containing protein	27	7	286	31.5	5.24	1.345	0.0212	35.1
227.	A0A0R0LB64	Uncharacterized protein	68	13	319	34.2	9.29	1.343	0.0038	34.19.1
228.	I1M841	ATPase AAA core domain-containing protein	34	12	478	52.4	5.96	1.343	0.0284	1.3.13
229.	C6TIM5	Ferredoxin--NADP reductase, chloroplastic	56	14	377	42.2	8.32	1.34	0.0318	7.3
230.	A0A0R0I4T4	Uncharacterized protein	45	4	296	31.7	7.85	1.338	0.0351	34.19.1
231.	I1K693	Uncharacterized protein	43	22	610	68.3	7.99	1.336	0.0339	30.5
232.	K7KZN7	Uncharacterized protein	19	17	1185	129.9	7.96	1.331	0.0174	10.8.1
233.	P08170	Seed linoleate 13S-lipoxygenase-1	85	57	839	94.3	6.39	1.328	0.0068	17.7.1.2
234.	I1J6K4	Uncharacterized protein	45	27	928	102.9	6.7	1.327	0.0064	26.3.1
235.	I1K6M2	Uncharacterized protein	52	9	222	23.8	7.87	1.326	0.0351	20.2
236.	A0A0R4J3L4	Usp domain-containing protein	36	8	255	27.4	6.3	1.323	0.0166	20.2.99
237.	C6SXY4	UBIQUITIN CONJUGAT 2 domain-containing protein	42	3	167	18.7	5.19	1.323	0.0400	29.5.11.3
238.	A0A0R0JYY8	Tubulin beta chain	72	24	446	49.9	5.01	1.32	0.0400	31.1
239.	I1MWQ0	Uncharacterized protein	37	11	366	39.8	7.01	1.309	0.0112	35.2
240.	A0A0R0GWM0	FAS1 domain-containing protein	40	8	250	26.6	8.54	1.296	0.0169	10.5.1
241.	I1L4U2	Serine hydroxymethyltransferase	65	21	517	57	7.59	1.294	0.0191	25.1
242.	C6TFW4	Bet v 1 domain-containing protein	43	7	158	16.7	5.05	1.277	0.0297	20.2.99
243.	I1J8T0	Isoflavone reductase	56	13	318	35.6	5.47	1.272	0.0393	16.8.5
244.	I1MFN6	Uncharacterized protein	75	8	167	17.9	3.93	1.265	0.0406	#N/A
245.	A0A0R0JCW2	Uncharacterized protein	43	18	583	66.2	6.29	1.264	0.0426	#N/A
246.	A0A0R0HF66	CSD 1 domain-containing protein	30	4	212	21	4.73	1.264	0.0429	27.3.75

247.	C6T019	Stress-response A/B barrel domain-containing protein	81	7	105	11.8	5.77	1.261	0.0457	35.2
248.	Q53B72	Chalcone-flavonone isomerase family protein	80	10	209	23.5	4.92	0.798	0.0485	16.8.2
249.	C6TER4	NmrA domain-containing protein	43	10	312	34.9	6.81	0.782	0.0473	16.2
250.	C6SWN5	Uncharacterized protein	28	4	120	13.7	11.6	0.774	0.0159	29.2.1.2.2.34
251.	C6SWV3	Uncharacterized protein	76	12	245	27.6	6.1	0.772	0.0145	35.2
252.	C6SVL1	Ribosomal protein	60	14	216	24.6	9.82	0.771	0.0393	29.2.1.1.3.2.510
253.	I1MYB0	Uncharacterized protein	74	21	484	53.4	6.09	0.767	0.0113	15
254.	A0A0R0GZ36	Uncharacterized protein	55	10	377	41.3	5.99	0.764	0.0347	35.1
255.	A0A0R0F2G6	Uncharacterized protein	55	19	482	52.5	7.96	0.761	0.0233	13.2.4.1
256.	A0A0R0F392	Cysteine synthase	27	9	372	40.1	7.14	0.748	0.0192	13.1.5.3.1
257.	C6SYW6	40S ribosomal protein S27	24	2	86	9.6	8.73	0.739	0.0077	29.2.1.2.1.27
258.	I1L3Q2	BOWMAN BIRK domain-containing protein	21	2	109	12.1	5.44	0.739	0.0297	#N/A
259.	I1KNN1	Uncharacterized protein	48	5	158	17.6	10.4	0.738	0.0324	29.2.1.2.1.15
260.	A0A0R0GXL3	MLO-like protein	15	4	539	61.3	10.45	0.73	0.0198	20.1.3.1
261.	I1JFL5	Inhibitor I9 domain-containing protein	32	4	136	14.7	6.52	0.729	0.0224	35.1
262.	C6T336	Uncharacterized protein	39	7	135	15.7	9.98	0.724	0.0012	29.2.1.2.1.17
263.	I1N8I7	Uncharacterized protein	25	3	149	16.9	4.28	0.722	0.0054	30.3
264.	I1JW21	Uncharacterized protein	48	7	143	16	10.24	0.713	0.0156	29.2.1.2.1.19
265.	I1MW04	Protein kinase domain-containing protein	8	3	360	40.9	4.89	0.713	0.0004	29.4
266.	I1MRJ8	Uncharacterized protein	20	11	722	78.9	8.68	0.71	0.0307	11.9.4.9
267.	I1LBB9	Lipoxygenase	26	16	865	98.1	5.92	0.707	0.0104	17.7.1.2
268.	I1M5Q9	Uncharacterized protein	38	11	374	40.9	7.99	0.704	0.0070	35.2
269.	C6TAW2	40S ribosomal protein S24	26	3	137	15.8	10.64	0.697	0.0014	29.2.1.2.1.24
270.	I1JJC4	Uncharacterized protein	11	4	391	43.6	7.91	0.694	0.0156	23.1.1.2
271.	I1K0S0	Ribosomal protein L15	41	8	204	24.2	11.59	0.692	0.0035	29.2.1.2.2.15
272.	F7J077	Beta-conglycinin beta subunit 2	27	6	439	50.4	6.24	0.687	0.0458	33.1
273.	A0A0R0F139	PKS ER domain-containing protein	32	7	361	39.3	7.28	0.686	0.0028	16.2.1.10
274.	A0A0R4J4L3	Annexin	41	14	312	35.9	7.91	0.685	0.0005	31.1
275.	I1J7H3	Ferritin	37	7	257	28.7	6.18	0.679	0.0019	15.2
276.	A0A0R0F214	U6 snRNA-associated Sm-like protein LSm4	9	2	165	18.9	5.27	0.669	0.0002	27.1
277.	I1KBE3	Alba domain-containing protein	28	4	241	27	10.13	0.657	0.0062	27.3.67

278.	I1NA10	Uncharacterized protein	31	8	359	38.8	6.65	0.656	0.0000	13.1.3.5.4
279.	K7KIP5	X8 domain-containing protein	8	3	489	53.4	6.25	0.655	0.0349	26.4
280.	C6SXI6	40S ribosomal protein S26	50	6	130	14.9	10.9	0.652	0.0062	29.2.1.2.1.26
281.	Q9SWB2	Seed maturation protein PM41	35	2	78	8.2	4.97	0.65	0.0076	35.2
282.	I1KXC2	PPDK N domain-containing protein	28	28	1459	163.7	6.84	0.646	0.0035	2.2.2.3
283.	I1L957	Uncharacterized protein	42	16	449	48.8	6.42	0.646	0.0000	33.2
284.	A0A0R0LK39	Uncharacterized protein	36	5	249	27.2	6.89	0.646	0.0002	20.2
285.	I1MUZ3	AAI domain-containing protein	16	2	178	18.9	8.76	0.645	0.0022	26.21
286.	I1KY16	Uncharacterized protein	23	4	320	35.5	6.44	0.642	0.0073	23.1.3
287.	A0A0R4J3J1	Peroxidase	42	10	324	35.2	8.06	0.64	0.0000	20.2.2
288.	A0A0R0KSS1	PKS ER domain-containing protein	24	7	343	37.9	6.27	0.638	0.0023	26.7
289.	I1KIS2	Aldedh domain-containing protein	11	4	501	54.6	5.67	0.633	0.0336	5.1
290.	I1MEF8	Clathrin light chain	28	8	322	35.3	5.19	0.631	0.0346	35.2
291.	I1KWD8	Uncharacterized protein	10	2	451	49.8	5.27	0.611	0.0271	26.2
292.	I1N407	Uncharacterized protein	18	4	297	32.7	6.7	0.608	0.0183	35.2
293.	I1KY36	TPRREGION domain-containing protein	19	6	482	54.5	6.09	0.601	0.0071	29.4
294.	A0A0R0EX12	Histone H4	33	5	156	17.9	11.36	0.6	0.0000	31.1
295.	C6TJP8	Uncharacterized protein	21	3	303	32.2	5.27	0.595	0.0054	29.5.11.20
296.	C6SXM8	Ribosomal protein L19	30	5	212	24.6	11.44	0.59	0.0076	29.2.1.2.2.19
297.	A0A0R0HEQ3	Histone H2B	23	3	149	16.2	10.08	0.586	0.0000	28.1.3
298.	A0A0R0JPL6	Peptidase A1 domain-containing protein	44	10	468	50	8.24	0.579	0.0001	27.3.99
299.	Q9XET0	Seed maturation protein PM30	34	5	140	15.1	8.9	0.577	0.0000	33.2
300.	A0A0R4J3J0	Uncharacterized protein	73	17	365	39.8	5.66	0.576	0.0042	16.2.1.9
301.	I1L7F3	Ribosomal L14e domain-containing protein	39	7	132	15.4	10.46	0.575	0.0000	29.2.1.2.2.14
302.	Q9ZTY1	35 kDa seed maturation protein	11	3	316	35.3	6.32	0.566	0.0012	33.2
303.	C6SWA6	Histone H2A	46	4	149	15.7	10.67	0.559	0.0000	28.1.3
304.	K7LUZ1	Uncharacterized protein	12	3	287	33.2	8.68	0.558	0.0000	35.2
305.	C6SZA4	Histone H3	45	5	136	15.3	11.3	0.551	0.0000	28.1.3
306.	O23957	Dehydrin	34	3	166	17.3	9.23	0.551	0.0000	20.2.99
307.	I1MHL7	Pectinesterase	6	2	528	58.1	6.3	0.534	0.0245	10.8.1
308.	Q9S7N8	Seed maturation protein PM21	48	2	95	10.1	5.01	0.53	0.0002	35.2

309.	A0A0R4J3P1	Glutamine synthetase	60	15	356	39.1	5.48	0.523	0.0005	12.2.2
310.	I1L053	Uncharacterized protein	37	9	342	39.2	5.74	0.515	0.0306	16.1.2.9
311.	I1JLC8	Protein SLE2	49	5	105	11.5	5.6	0.515	0.0000	17.1.3
312.	K7M9Q3	Chlorophyll a-b binding protein, chloroplastic	25	3	260	27.6	6.6	0.507	0.0326	1.1.1.1
313.	I1NG96	J domain-containing protein	9	2	305	35.7	9.35	0.499	0.0003	20.2.1
314.	I1LJ14	Uncharacterized protein	13	7	770	81.5	9.04	0.486	0.0025	29.5.1
315.	K7KPN3	Uncharacterized protein	43	7	211	23.4	8.16	0.476	0.0014	30.5
316.	I1JPH6	Lambda class glutathione S-transferase	18	3	235	26.8	6.01	0.468	0.0000	26.9
317.	A0A0R0F3E0	Histone H2A	25	2	144	15.5	10.71	0.465	0.0000	28.1.3
318.	C6T197	Bap31 domain-containing protein	40	3	129	14.9	8.79	0.446	0.0000	35.2
319.	A0A368UH16	Aminotran 5 domain-containing protein	9	2	468	51.5	6.52	0.445	0.0000	30.1.1
320.	I1K554	Knot1 domain-containing protein	23	2	74	8.4	9.16	0.435	0.0000	20.1
321.	A0A0R0IH13	Uncharacterized protein	36	4	127	15	8.31	0.433	0.0000	9.1.2
322.	K7N485	Phenylalanine ammonia-lyase	16	8	717	78.3	6.98	0.405	0.0000	16.2.1.1
323.	A0A0R4J4J7	Uncharacterized protein	23	6	320	35.7	9.54	0.396	0.0031	16.7
324.	I1J4F4	Uncharacterized protein	6	2	626	67.5	6.49	0.396	0.0008	21.2.2
325.	K7LEQ5	Uncharacterized protein	19	3	253	26.6	6.8	0.395	0.0000	#N/A
326.	A0A0R4J3L3	NADPH-protochlorophyllide oxidoreductase	26	7	399	43	9	0.359	0.0011	19.14
327.	A0A0R0LFG1	Uncharacterized protein	2	2	1066	118.6	6.55	0.355	0.0013	29.3.1
328.	K7KVC7	Knot1 domain-containing protein	32	2	76	8.6	8.46	0.346	0.0000	20.1
329.	C6SWR8	Ribonucloprotein	66	4	128	13.8	7.12	0.342	0.0000	29.2.1.2.2.57
330.	K7KLV4	Uncharacterized protein	1	2	2392	269.9	5.36	0.342	0.0004	35.1
331.	A0A0R0FK13	Uncharacterized protein	9	2	422	46.5	6.6	0.301	0.0001	34.12
332.	I1JA20	MINDY DUB domain-containing protein	7	5	726	79.6	4.65	0.259	0.0000	35.2
333.	P11827	Beta-conglycinin alpha' subunit	22	10	621	72.2	5.71	0.238	0.0000	33.1
334.	A0A0R0HS93	Uncharacterized protein	7	10	1141	133.1	5.85	0.158	0.0000	29.5.11
335.	I1K1Z8	PCI domain-containing protein	24	8	423	47.6	6.55	0.113	0.0000	30.11.1
336.	A0A0R0EAL6	PKS ER domain-containing protein	52	13	357	39	5.8	0.11	0.0000	16.2.1.10
337.	A0A0R0EXW6	AAA domain-containing protein	58	17	446	49.5	6.21	0.01	0.0000	29.5.11.20
338.	A0A368UHI2	Uncharacterized protein	42	16	543	57.7	5.33	0.01	0.0000	20.2.1
339.	K7KYQ4	Uncharacterized protein	26	8	605	68	7.52	0.01	0.0000	27.1.1

340.	K7MUD9	AB hydrolase-1 domain-containing protein	23	7	520	58.2	7.83	0.01	0.0000	29.5
341.	I1M6R4	Uncharacterized protein	51	7	188	20.2	8.13	0.01	0.0000	27.1.19
342.	I1MV80	Cupin 2 domain-containing protein	47	6	293	33	6.61	0.01	0.0000	27.3.30
343.	I1K354	Uncharacterized protein	17	6	398	45.1	6.87	0.01	0.0000	23.1.1.2
344.	I1J575	Uncharacterized protein	35	5	259	28.8	8.51	0.01	0.0000	29.3.4.99
345.	A0A0R0JMD5	Uncharacterized protein	35	5	192	21.1	5.01	0.01	0.0000	29.5.11
346.	I1JJW5	Uncharacterized protein	10	4	549	61.5	5.83	0.01	0.0000	30.3
347.	I1KSL3	Uncharacterized protein	7	4	837	89.9	5.55	0.01	0.0000	29.4
348.	P0DO16	Beta-conglycinin alpha subunit 1	8	3	605	70.3	5.17	0.01	0.0000	33.1
349.	I1LNG0	Uncharacterized protein	7	2	552	63	8.85	0.01	0.0000	17.2.3
350.	I1KU65	Uncharacterized protein	7	2	369	40.3	6.87	0.01	0.0000	26.7
351.	I1KZY8	Uncharacterized protein	6	2	536	59	5.12	0.01	0.0000	27.2

[a)] Matched peptide, [b)] amino acids, [c)] molecular weight, [d)] calculated isoelectric point, [e)] MapMan bin codes taken from https://mapman.gabipd.org/
